# Longitudinal Plasma Ferritin in the First Year of Life in Relation to Maternal Status, Birth Characteristics, and Breastfeeding

**DOI:** 10.3390/nu18111657

**Published:** 2026-05-22

**Authors:** Mia Stråvik, Inger-Cecilia Mayer Labba, Malin Barman, Linnéa Svärd, Nathalie Scheers, Anna Sandin, Agnes E. Wold, Ann-Sofie Sandberg

**Affiliations:** 1 Department of Life Sciences, Food and Nutrition Science, Chalmers University of Technology, 412 96 Gothenburg, Sweden; mia.stravik@chalmers.se (M.S.); malin.barman@chalmers.se (M.B.); lsvard@student.chalmers.se (L.S.); nathalie.scheers@chalmers.se (N.S.); ann-sofie.sandberg@chalmers.se (A.-S.S.); 2 Sunderby Research Unit, Department of Clinical Science, Pediatrics, Umeå University, 901 87 Umeå, Sweden; anna.sandin@umu.se; 3 Department of Infectious Diseases, Institute of Biomedicine, Sahlgrenska Academy, University of Gothenburg, 405 30 Gothenburg, Sweden; agnes.wold@microbio.gu.se

**Keywords:** ferritin, iron, infancy, childhood, birth characteristics, cohort, cord blood

## Abstract

**Background/Objectives**: Iron deficiency early in life can impair infant growth and cognitive development. Here, we follow infants’ plasma ferritin levels—an indicator of iron stores—over the first year of life and relate these to birth characteristics, maternal characteristics, and infant feeding. **Methods**: Children and their mothers enrolled in the Swedish birth cohort NICE (ClinicalTrials.gov identifier: NCT05809479) were followed from pregnancy to twelve months postpartum. Plasma ferritin was quantified in umbilical cord blood at birth (*n* = 345), in venous plasma at four months after birth (mother–infant dyads, *n* = 133), and at twelve months of age (*n* = 158), using sandwich ELISA. Perinatal and postnatal growth, together with infant and maternal characteristics, were extracted from medical birth records. Breastfeeding and formula feeding were assessed using repeated monthly questionnaires during the first year. Longitudinal changes were analyzed using linear mixed-effects models, and factors associated with ferritin concentrations were examined using Spearman correlations, linear regression models, and segmented generalized additive models. **Results**: The ferritin concentration declined over time (birth: 267 ng/mL; four months: 146 ng/mL; twelve months: 30 ng/mL). Boys had lower ferritin levels than girls at all timepoints. Ferritin status at four and twelve months was positively associated with ferritin concentrations in cord blood and with gestational age. Breastfeeding and formula feeding were not associated with ferritin concentrations. **Conclusions**: Infant sex, cord ferritin concentrations, and maternal ferritin concentrations were independently associated with infant ferritin concentrations across the first year of life, whereas neither breastfeeding nor formula feeding was associated with ferritin concentrations in the present analyses. Infant sex, cord ferritin, and maternal ferritin measured four months postpartum may help identify children at risk of low iron stores, with maternal ferritin potentially offering a less intrusive alternative to repeated infant sampling. However, the clinical relevance and potential use of maternal ferritin as a proxy for infant ferritin concentrations require further investigation.

## 1. Introduction

Iron deficiency is the most prevalent micronutrient deficiency worldwide and, when occurring in early life, has been associated with impaired neurodevelopment [1,2,3]. Infancy is a particularly vulnerable period because of rapid growth and expanding blood volume, which lead to high iron requirements [3,4].

Fetal iron stores are established in utero and depend on placental transfer of iron from the mother, with substantial accumulation occurring late in gestation [5,6]. Preterm birth is therefore associated with suboptimal iron stores and an increased risk of iron deficiency [7].

After birth, infant iron stores are progressively depleted to support growth and erythropoiesis, and dietary iron intake becomes increasingly important [1,4]. Perinatal factors (e.g., maternal iron status, gestational age, birth weight, and infant sex) and early feeding practices (breastfeeding and infant formula use) may influence iron status in infancy [1,4,7,8]. Although breast milk contains relatively low concentrations of iron, the bioavailability of this iron is high, with estimated absorption rates of approximately 37–70% [9,10,11]. In contrast, iron absorption from infant formula and other non-heme iron sources is generally lower [12,13]. Together with iron stores accumulated before birth, breast milk is therefore considered sufficient to meet infant iron requirements during the first four to six months of life [14,15].

Ferritin is widely used as a marker of iron status in clinical practice and epidemiological research, yet appropriate ferritin cut-offs and reference intervals for diagnosing iron deficiency in neonates and infants remain uncertain [16,17,18]. Existing cut-offs are largely derived from statistical distributions or associations with hemoglobin rather than validated physiological thresholds specific to early infancy [3,17,18,19]. In addition, although some data on ferritin is available from Nordic populations [8,17,20,21], longitudinal data from birth through the first year of life in healthy populations remain limited, particularly regarding maternal, perinatal, and early postnatal determinants of infant ferritin concentrations. Hence, a clearer understanding of ferritin patterns during infancy is important for identifying those at risk of inadequate iron status at an early stage.

Using data from a prospective Swedish birth cohort, the primary aim was to examine plasma ferritin levels at birth, four months, and twelve months of age. A secondary aim was to identify potential factors influencing infant iron status, including maternal plasma ferritin levels.

## 2. Materials and Methods

### 2.1. Study Population

This study is part of the Swedish birth cohort Nutritional impact on Immunological maturation during Childhood in relation to the Environment (NICE) (ClinicalTrials.gov identifier: NCT05809479, 12 April 2023, retrospectively registered). The NICE study followed families in northern Sweden from pregnancy week 18, when recruitment took place during a routine ultrasound, until the child reached six years of age. All expecting parents with planned delivery at Sunderby Hospital in Norrbotten County, and with the ability to communicate in Swedish, were invited to participate. A total of 655 pregnancies were successfully enrolled during the recruitment period from February 2015 to March 2018. More detailed information about the cohort can be found in the study protocol [22].

**Figure 1:**
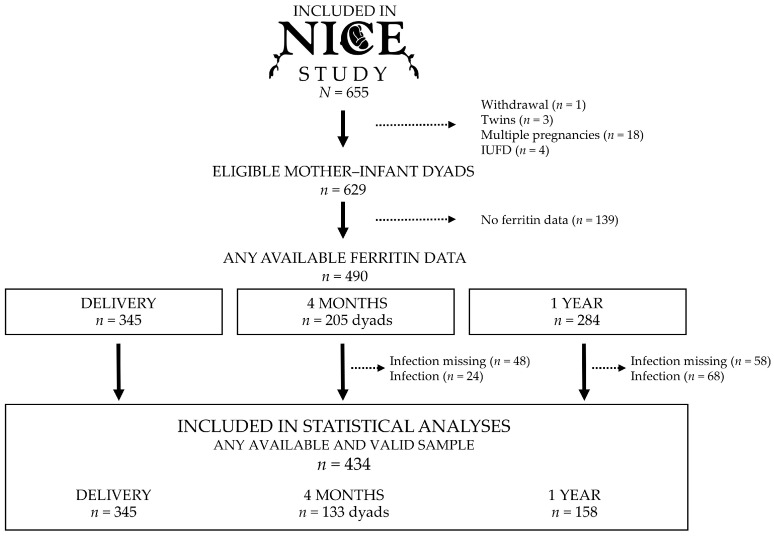
Flow chart of inclusion.

For this specific study of ferritin status, twins, second siblings within the NICE cohort, and stillbirths were excluded, resulting in 629 eligible mother–infant dyads. Among these, 490 children had at least one available ferritin measurement at birth, four months, or twelve months (*n* = 345 infants at birth; *n* = 205 dyads at four months; *n* = 284 children at twelve months). In a second step, ferritin samples at four and twelve months were kept only if parents actively reported no infection at the time of sampling. Hence, a total of 434 children were included in the statistical analyses of this study (*n* = 345 infants at birth; *n* = 133 mother–infant dyads without reported infection at four months; *n* = 158 children without reported infection at twelve months) (Figure 1). Comparisons of characteristics between included and excluded children and their mothers are presented in Supplementary Table S1 (excluded due to infection and/or missing infection information) and Supplementary Table S2 (excluded due to missing ferritin data, infection, and/or missing infection information).

### 2.2. Sample Collection and Analysis of Plasma Ferritin

Blood samples were collected at three timepoints: birth (child), four months after birth (mother and child), and twelve months of age (child). At birth, blood was collected from the umbilical cord. More specifically, the umbilical cord was clamped immediately after birth, and the blood was squeezed into 6 mL EDTA tubes (Becton, Dickinson and Company, Franklin Lakes, NJ, USA).

Four months after birth, venous samples were collected from the children (dorsal side of the hand) into 3 mL EDTA tubes (Becton, Dickinson and Company, Plymouth, UK), and from their mothers (cubital vein) into 10 mL EDTA tubes (Becton, Dickinson and Company, Franklin Lakes, NJ, USA).

At twelve months of age, venous samples were collected from the child into 5 mL EDTA tubes (Becton, Dickinson and Company, Franklin Lakes, NJ, USA) during a follow-up visit at the study clinic.

Samples from all timepoints were temporarily stored at 4 °C until centrifugation at the hospital laboratory for 5 min at 2400 rpm (Hettich Rotina 420, Hettich Lab Technology, Tuttlingen, Germany) to separate the plasma. After centrifugation, all samples were transferred to −80 °C for long-term storage. Detailed procedures have been published previously [22,23].

Ferritin concentrations were measured by sandwich ELISA (Ferritin ELISA kit EIA-4408; DRG Instruments GmbH, Marburg, Germany) following the manufacturer’s protocol.

### 2.3. Assessment of Infection and Covariates

As ferritin is an acute-phase protein sensitive to infection and inflammation, plasma samples potentially affected by concurrent infections were excluded. Information about infant infections was collected via monthly parental questionnaires during the first year of life. More specifically, parents answered yes/no to: “Has your child had an infectious disease during the past month?”. If yes, follow-up questions captured infection type, days with fever above 38.5 °C, and treatment received (if any). Plasma samples were included only if ferritin concentrations were available and paired with a negative response to the infection question at four months and/or twelve months. Infant infections served as a proxy for maternal infections at four months. Umbilical cord samples at birth were not excluded based on infection. Characteristics of included children and children excluded due to infection or missing infection information are presented in Supplementary Table S1.

Information on maternal characteristics (age, education, body mass index in early pregnancy, and parity), delivery characteristics (mode of delivery, gestational age, and preterm birth), and infant characteristics (birth weight, small-for-gestational-age [24] large-for-gestational-age, birth length, and sex) were extracted from hospital records. Small-for-gestational-age and large-for-gestational-age were defined using both percentile-based cut-offs (below the 10th and above the 90th percentiles, respectively) and standard deviation-based cut-offs (below −2 and above +2 standard deviations, respectively), according to ultrasound-derived, sex-specific growth curves from Maršál et al. [24]. Children’s weight and height at one year of age were collected via a semi-quantitative food frequency questionnaire administered when the child turned one year. Information on breastfeeding and formula feeding during the first year was collected using monthly questionnaires covering the preceding month.

### 2.4. Statistical Analysis

All analyses were performed using IBM SPSS Statistics version 28 (IBM Corp., Armonk, NY, USA) and R version 4.5.1 (R Foundation for Statistical Computing, Vienna, Austria). For all analyses, *p* values < 0.05 were considered statistically significant.

#### 2.4.1. Longitudinal Analysis

Ferritin concentrations were log_2_-transformed because of right-skewed distributions, as assessed by histograms and Shapiro–Wilk tests. Children with at least two ferritin measurements (i.e., data available for two or more of the three timepoints: birth, four months, and twelve months) were included in this longitudinal analysis (*n* = 167). Longitudinal changes in ferritin concentrations were analyzed using linear mixed-effects models (LMMs) with log_2_-transformed ferritin concentration as the dependent variable, fitted using the *lme4* package (version 1.1.37) in R [25]. Timepoint (birth, four months, and twelve months) was included as a categorical fixed effect because ferritin concentration was measured at three discrete visits, and birth was used as the reference category. A random intercept for each child was included to account for within-child correlation of repeated measurements. A timepoint × sex interaction was tested using a likelihood ratio test (LRT), comparing a reduced model including timepoint and sex as main effects with a full model including the timepoint × sex interaction. Both models were fitted using maximum likelihood, and the interaction results were presented together with sex-stratified analyses. Regression coefficients (four months versus birth, and twelve months versus birth) and the model-estimated pairwise difference (twelve months versus four months) were presented as β, together with 95% confidence intervals. The pairwise comparison was obtained using the *emmeans* package (version 1.11.2.8) in R [26]. For all comparisons, a one-unit difference corresponds to a two-fold difference in ferritin concentration. The intraclass correlation coefficient (ICC) was calculated to describe the proportion of total variance in ferritin concentrations explained by between-child differences. For visualization, predicted means with 95% confidence intervals and individual ferritin measurements over time were plotted, with the *y*-axis on the log_2_ scale and the *x*-axis scaled to reflect the sampling times (birth, four months, and twelve months).

#### 2.4.2. Exploratory Analysis of Factors Associated with Ferritin Concentrations

Plasma ferritin concentrations at birth, four months, and twelve months were examined in relation to a priori selected covariates using an exploratory approach followed by linear regression modeling. The covariates included: maternal age (continuous, years), maternal education (categorical, 1 = Elementary school, 9 years; 2 = High school, 12 years; and 3 = University or other, >12 years), maternal body mass index in early pregnancy (BMI; continuous, kg/m^2^), parity (binary, 0 = first child, 1 = one or more previous children), mode of delivery (binary, vaginal = 0, caesarean section = 1), gestational age (continuous, days), preterm birth (binary, 0 = term, 1 = preterm i.e., born before gestational day 258 or 36 + 6 weeks), birth weight (continuous, g), birth length (continuous, cm), small-for-gestational-age (SGA; binary, 0 = no, 1 = yes), large-for-gestational-age (LGA; binary, 0 = no, 1 = yes), sex (binary, 0 = female, 1 = male), weight at one year of age (continuous, kg), height at one year of age (continuous, cm), breastfeeding duration (continuous, months), formula feeding duration (continuous, months), umbilical cord ferritin concentrations (continuous, ng/mL), ferritin concentrations at four months of age (continuous, ng/mL), maternal ferritin concentrations at four months postpartum (continuous, ng/mL), and ferritin concentrations at twelve months of age (continuous, ng/mL). To note, associations lacking biological plausibility were not investigated (i.e., umbilical cord ferritin in relation to maternal ferritin at four months, breastfeeding duration, or formula feeding duration).

In the exploratory step, Spearman correlation analyses between raw ferritin concentrations and potential covariates were conducted and visualized in a heatmap using the *pheatmap* package (version 1.0.13) in R [27]. To account for multiple testing, *p* values were adjusted for the false discovery rate (FDR) using the Benjamini–Hochberg procedure.

In a secondary step, crude and multivariable linear regression models were fitted using log_2_-transformed ferritin concentrations at birth, four months, and twelve months as outcomes (separate models per timepoint and exposure). Exposures were selected based on associations from the exploratory Spearman analyses with FDR-adjusted *p* values < 0.05. All multivariable models were adjusted for infant sex (Model 2) and infant sex and gestational age (Model 3).

Previous research has indicated non-linear relationships between gestational age at birth, birth weight, and umbilical cord blood ferritin concentrations [28]. Therefore, secondary analysis of non-linear relationships was examined using segmented linear regression. The presence and location of a breakpoint were assessed using the *segmented* package (version 2.2.1) [29]. Thereafter, generalized additive models (GAMs) with smoothing splines were fitted to data below and above, respectively, the identified breakpoints using the *mgcv* package (version 1.9.3) [30]. The GAM curves with 95% confidence intervals were visualized using *ggplot2* package (version 3.5.2) [31].

## 3. Results

The background characteristics of the included children and their mothers are presented in Table 1. The children had a median birth weight of 3565 g and a median birth length of 50 cm. The majority were born vaginally (89%), and 5% were born preterm. Depending on the definition used (percentiles or standard deviations), 2–8% were classified as small for gestational age and 4–14% as large for gestational age.

Among the 400 children with any response to the monthly questionnaires from the first year of life, 18 (<5%) were never breastfed. Breastfeeding continued up to a median of eight months of age, and at four months, 64% of the children were exclusively breastfed. A total of 75 children (19%) were breastfed up to twelve months of age. The mothers had a median age of 30 years, and the majority had a higher education (71% had studied for more than twelve years).

Comparisons of characteristics between included and excluded children and their mothers are presented in Supplementary Table S1 (excluded due to infection and/or missing infection information) and Supplementary Table S2 (excluded due to missing ferritin data, infection, and/or missing infection information).

### 3.1. Descriptive Analysis of Ferritin Concentrations

Ferritin concentrations were measured at birth, four months, and twelve months of age and the respective median concentrations are presented in Table 2. As can be seen, the median concentrations decreased for each timepoint, starting at 267 ng/mL at birth, followed by 146 ng/mL at four months, and 30 ng/mL at twelve months of age. At four months postpartum, maternal median ferritin concentration was 39 ng/mL, corresponding to 27% of the infant median concentration at the same timepoint. For context, Table 2 also presents the proportions of participants with ferritin levels below commonly used thresholds [16,32,33], although such thresholds vary across the literature.

To note, the ferritin concentrations among the children and their mothers excluded due to self-reported infection, or lack of that information, were higher at four months for both the children (172 ng/mL versus the included children with 146 ng/mL) and the mothers (44 ng/mL versus 39 ng/mL), but similar at twelve months (29 ng/mL versus 30 ng/mL) (Supplementary Table S3).

**Figure 2:**
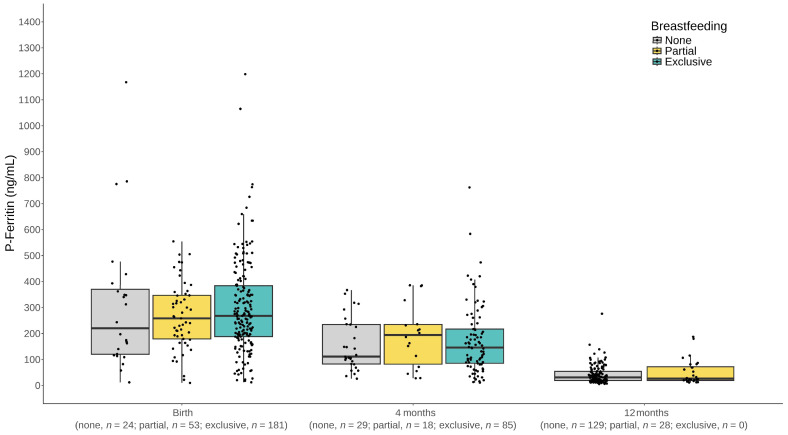
Infant plasma ferritin concentrations by breastfeeding extent (none, partial, exclusive) at birth, four months, and twelve months of age. Differences between groups were assessed using the Kruskal–Wallis test. No statistically significant differences were observed.

Ferritin concentrations did not differ by breastfeeding extent (not at all, partially, exclusively) or formula feeding extent at any of the sampling occasions (Kruskal–Wallis test, non-significant; Figure 2 and Supplementary Table S4, respectively).

**Figure 3:**
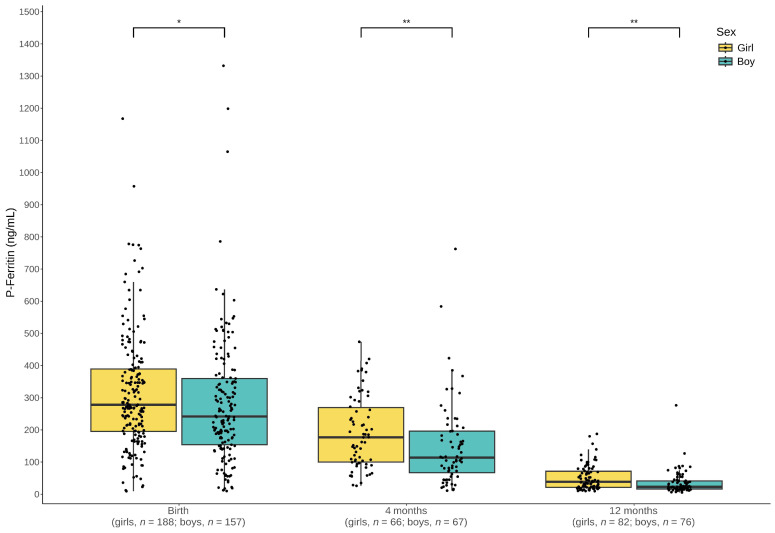
Infant plasma ferritin concentrations by sex at birth, four months, and twelve months of age. Differences were tested using the Mann–Whitney U test. Statistical significance is denoted as: *** = *p* < 0.001, ** = *p* < 0.01, * = *p* < 0.05, and NS = *p* ≥ 0.05.

In Figure 3, differences in ferritin concentrations between boys and girls are visualized. As can be seen, girls had significantly higher plasma ferritin concentrations than boys at all timepoints: birth (278 versus 242 ng/mL, *p* = 0.030), four months (177 versus 114 ng/mL, *p* = 0.009), and twelve months of age (39 versus 23 ng/mL, *p* = 0.001).

### 3.2. Longitudinal Analysis of Ferritin Concentrations

**Figure 4:**
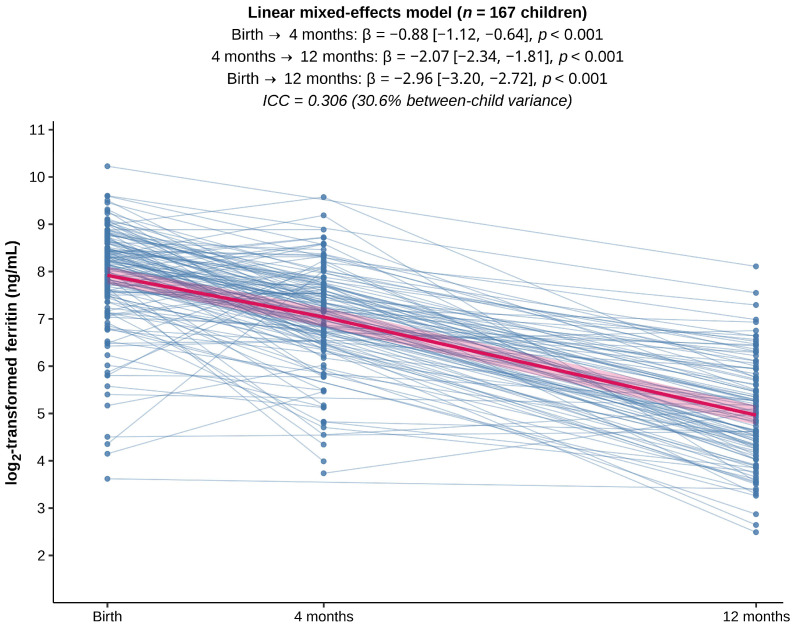
Longitudinal analysis of infant plasma ferritin concentrations over the first year of life. Blue lines show individual children’s repeated measures; the red line and shaded area indicate the model-based population mean ± 95% CI. Regression coefficients for four months versus birth and twelve months versus birth, as well as the model-estimated pairwise difference between twelve and four months, are presented as β.

In a linear mixed-effects model (*n* = 167 children with at least two repeated measurements), log_2_-transformed ferritin concentrations were lower at four months and twelve months than at birth (four months: β [95% CI] = −0.88 [−1.12, −0.64], *p* < 0.001; twelve months: β [95% CI] = −2.96 [−3.20, −2.72], *p* < 0.001), and were also lower at twelve months than at four months (β [95% CI] = −2.07 [−2.34, −1.81], *p* < 0.001) (Figure 4). On the log_2_ scale, one β unit corresponds to a two-fold difference in ferritin concentration. Thus, the ferritin concentrations at four months were approximately 0.54-fold those at birth (2^−0^·^88^ ≈ 0.54; 46% lower), and concentrations at twelve months were approximately 0.13-fold those at birth (2^−2.96^ ≈ 0.13; 87% lower). The intraclass correlation coefficient (ICC) was 0.306, indicating that 30.6% of the total variance in ferritin concentrations was due to between-child differences.

In sex-stratified analyses (Supplementary Figure S1), boys had slightly larger difference in ferritin concentrations between birth and twelve months (boys: β [95% CI] = −3.14 [−3.53, −2.75], *p* < 0.001; girls: β [95% CI] = −2.80 [−3.09, −2.51], *p* < 0.001), particularly between birth and four months (boys: β [95% CI] = −1.08 [−1.46, −0.71], *p* < 0.001; girls: β [95% CI] = −0.67 [−0.97, −0.36], *p* < 0.001). The estimated differences between four and twelve months were similar in boys and girls, although slightly larger in girls (boys: β [95% CI] = −2.06 [−2.47, −1.64], *p* < 0.001; girls: β [95% CI] = −2.13 [−2.46, −1.80], *p* < 0.001). However, a likelihood ratio test (LRT) comparing an LMM with main effects for timepoint and sex with a corresponding model additionally including a time × sex interaction showed no significant interaction (χ^2(df = 2)^ = 3.68, *p* = 0.159), indicating that the longitudinal declining pattern was similar in boys and girls. The ICC was higher in girls than in boys (boys: 0.269; girls: 0.342), suggesting that a larger proportion of the total variance in ferritin concentrations was due to between-child differences among girls (i.e., girls tended to maintain their relative ranking in ferritin concentrations to a greater extent over time than boys).

### 3.3. Factors Associated with Ferritin Concentrations

**Figure 5:**
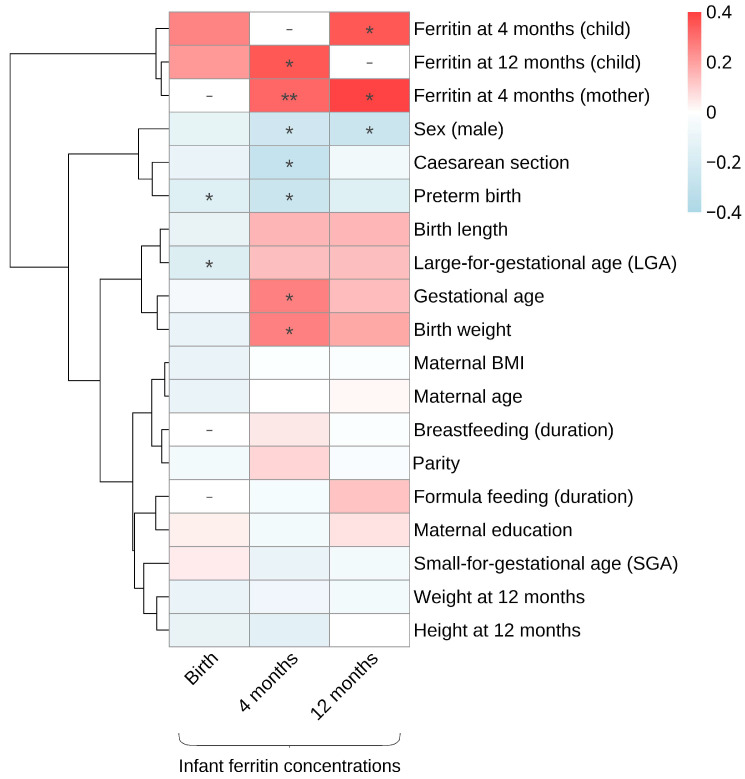
Spearman correlations between cross-sectional ferritin concentrations and potential influencing factors. The color indicates the direction and magnitude of each correlation coefficient (rho). Cells marked “–“ indicate correlations that were not conducted because the exposure occurred after blood sampling. All *p* values were adjusted for false discovery rate (FDR) using the Benjamini–Hochberg procedure. Statistical significance is denoted as: *** = FDR-adjusted *p* < 0.001, ** = FDR-adjusted *p* < 0.01, and * = FDR-adjusted *p* < 0.05.

As a first exploratory approach, associations between family and infant characteristics and plasma ferritin concentrations were investigated using Spearman correlation analyses (Figure 5). Variables that remained statistically significant after FDR adjustment (FDR-adjusted *p* values < 0.05) were considered of interest and were further investigated using linear regression models (Table 3).

#### 3.3.1. Correlation Analyses and Linear Regression Models

Cord blood ferritin concentrations were negatively associated with being born preterm (rho = −0.16, FDR-adjusted *p* = 0.023) and LGA (rho = −0.17, FDR-adjusted *p* = 0.019). Further, although the statistical significance did not remain after adjustment for multiple testing, cord blood ferritin concentrations showed positive associations with the two later sampling occasions (four months: rho = 0.26, FDR-adjusted *p* = 0.050; and twelve months: rho = 0.21, FDR-adjusted *p* = 0.123).

Plasma ferritin concentrations at four months of age were significantly associated with several of the a priori selected covariates. More specifically, positive associations were seen with gestational age (rho = 0.27, FDR-adjusted *p* = 0.019), birth weight (rho = 0.26, FDR-adjusted *p* = 0.019), ferritin concentrations at twelve months of age (rho = 0.35, FDR-adjusted *p* = 0.044), and with maternal ferritin concentrations at four months (rho = 0.32, FDR-adjusted *p* = 0.009). Negative associations were found with being a boy (rho = −0.23, FDR-adjusted *p* = 0.044), being delivered with caesarean section (rho = −0.28, FDR-adjusted *p* = 0.019), and being born preterm (rho = −0.25, FDR-adjusted *p* = 0.023).

At twelve months of age, plasma ferritin concentrations were positively associated with both the infant and maternal ferritin concentrations (rho = 0.35, FDR-adjusted *p* = 0.044, and rho = 0.39, FDR-adjusted *p* = 0.023, respectively) and negatively associated with being a boy (rho = −0.26, FDR-adjusted *p* = 0.019).

To further investigate factors associated with ferritin levels, linear regression models were fitted for ferritin concentrations at earlier timepoints, maternal ferritin at four months postpartum, infant sex, caesarean section, preterm birth, LGA, gestational age (weeks), and birth weight (per 100 g), and adjusted for infant sex and gestational age (Table 3). Ferritin concentrations at birth were significantly associated with the ferritin concentrations at both four months (β = 0.29, *p* = 0.005; 22% higher concentrations per doubling) and twelve months (β = 0.31, *p* = 0.005; 24% higher concentrations per doubling). However, maternal ferritin concentrations were even more strongly associated with infant concentrations at four and twelve months (β = 0.33, *p* < 0.001; 25% higher concentrations per doubling; and β = 0.31, *p* = 0.010; 24% higher concentrations per doubling, respectively).

The factors most strongly associated with umbilical cord ferritin concentrations were preterm birth (β = −1.31, *p* = 0.001; 40% of the concentrations of term infants), male sex (β = −0.27, *p* = 0.023; 83% of the concentrations of girls), and being born LGA (β = −0.32, *p* = 0.049; 80% of the concentrations in non-LGA infants). To note, infant sex was strongly and independently associated with lower ferritin concentrations at all timepoints (birth: β = −0.27, *p* = 0.023; four months: β = −0.66, *p* = 0.001; and twelve months: β = −0.61, *p* < 0.001).

At four months of age, in addition to maternal ferritin concentrations, cord ferritin concentrations, and infant sex, the factors most strongly associated with ferritin concentrations were gestational age (β = 0.27 per week, *p* < 0.001; 21% higher concentrations per week) and caesarean section delivery (β = −1.00, *p* = 0.001; 50% of the concentrations of children born vaginally).

Ferritin concentrations at twelve months were most strongly associated (in addition to infant sex, infant ferritin levels at birth, and maternal ferritin levels at four months) with gestational age (β = 0.11 per week, *p* = 0.011; 8% higher concentrations per week) and birth weight (β = 0.04 per 100 g, *p* = 0.030; 3% higher concentrations per 100 g).

#### 3.3.2. Non-Linear Effects of Gestational Age and Birth Weight

**Figure 6:**
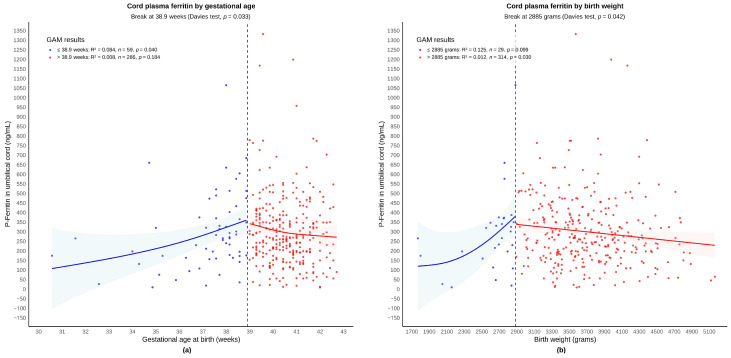
Segmented generalized additive models (GAMs) of gestational age and birth weight in relation to umbilical cord blood ferritin concentrations. (**a**) Gestational age at birth with a data-driven breakpoint at 38.9 weeks, and (**b**) birth weight with a data-driven breakpoint at 2885 g. Separate regression lines are shown for observations below and above each breakpoint, with shaded areas indicating the 95% confidence intervals.

As shown in Figure 6a, there was no strong overall linear association between gestational age and ferritin concentrations in umbilical cord blood. Since the literature suggests that the relationship may not be linear [28], we conducted secondary exploratory analyses using segmented regression analysis. For gestational age, a data-driven breakpoint at 38.9 weeks was identified. Below this point, ferritin concentrations increased with advancing gestation (≤38.9 weeks: R^2^ = 0.084, *n* = 59, *p* = 0.040), whereas no significant association was observed beyond it (>38.9 weeks: R^2^ = 0.008, *n* = 286, *p* = 0.184). This pattern, although it should be interpreted with caution due to the limited sample size below the estimated breakpoint, suggests that cord ferritin concentrations increase until late term and then plateau.

A similar pattern was observed for birth weight (Figure 6b). The segmented model identified a data-driven breakpoint at 2885 g. Among infants weighing ≤2885 g, ferritin concentrations tended to increase with increasing birth weight (R^2^ = 0.125, *n* = 29, *p* = 0.099), whereas among infants weighing >2885 g, the association was weak but statistically significant (R^2^ = 0.012, *n* = 314, *p* = 0.030). Given the limited number of infants in the lower birth weight subgroup, these results should be interpreted with caution. To note, gestational age and birth weight were strongly correlated (rho = 0.52, *p* < 0.001), likely reflecting the close relation between continuous fetal growth in utero and advancing gestation.

## 4. Discussion

In this longitudinal study of Swedish children, we observed significant ferritin declines over time, with boys consistently having lower levels than girls. Almost one-third of the ferritin variation was explained by between-infant differences originating already at birth. Ferritin declined significantly across all three timepoints while relative differences between infants remained consistent. Breastfeeding and formula feeding were not associated with ferritin concentrations.

Infant sex, umbilical cord ferritin, and maternal ferritin levels were the most evident factors associated with infant ferritin concentrations across the first year of life. At birth, gestational age and birth weight (strongly related to gestational age) showed weak, non-linear associations with umbilical cord ferritin levels. Given the strong association between gestational age and birth weight, the association between birth weight and ferritin concentrations should be interpreted with caution. Our results regarding umbilical cord ferritin align with prior work showing that fetal iron stores are progressively utilized to support rapid postnatal growth, and that infants who begin life with lower iron stores remain at elevated risk of low ferritin later in infancy [4,17,34].

The differences we found in ferritin concentrations between boys and girls are supported by previous studies consistently reporting higher levels among young girls [8,17,35], a trend that reverses completely from puberty onward [36]. Although differences have previously been reported, the clinical relevance of sex differences during infancy remains uncertain. More specifically, it is unclear whether boys can maintain adequate biological function at lower ferritin concentrations or if they are indeed more vulnerable to lower iron storage. Hence, although we found large differences between boys and girls, it remains unclear whether sex-specific thresholds for ferritin status would improve clinical assessment.

Gestational age and birth weight showed weak non-linear associations with umbilical cord ferritin. Although these results must be interpreted with caution due to the limited strength of the associations and the sample size in the subgroups, they may indicate that fetal iron accumulation increases up to around gestational week 39 and a birth weight of 2885 g before reaching a plateau. Preterm infants had lower ferritin concentrations both at birth and at four months, consistent with previous studies linking preterm birth to lower iron stores [14,37,38].

Interpreting the clinical relevance of ferritin concentrations during infancy remains challenging. Studies in Nordic populations investigating neurodevelopmental consequences of suboptimal iron status are limited. However, a Norwegian study found that lower ferritin concentrations (<51 ng/mL) were associated with suboptimal gross motor scores at three to seven months [39]. In contrast, a Swedish randomized controlled trial found that delayed cord clamping improved infant iron status at four months, but did not affect neurodevelopment at twelve months [40,41]. Further complicating interpretation, commonly used cut-offs for iron deficiency are typically derived from population-based distributions rather than clinical outcomes such as neurodevelopmental impairment [3,17,18,19]. Thresholds vary widely across the literature, with no consensus. In our study, 4–7% of infants had cord ferritin concentrations below 40–65 ng/mL at birth, 2% had ferritin below 20 ng/mL at four months, and 3–8% had ferritin below 10–12 ng/mL at twelve months. In contrast, a Canadian cohort applied a higher cord blood ferritin cut-off of 76 ng/mL (compared with the 40–65 ng/mL thresholds discussed here) and reported that 26% of the newborns were classified below that threshold [28]. Applying the same threshold in our cohort did not markedly change the classification, with only two additional children classified below the cut-off, corresponding to a total of 7.5% of children below the threshold. The difference may partly reflect methodological variation, as the Canadian study had a smaller sample size (*n* = 46 compared with *n* = 345 in our study) and mainly collected cord blood from caesarean section deliveries, which in that study were associated with lower ferritin concentrations than vaginal deliveries. In our cohort, where the majority were born vaginally, cord ferritin concentrations were also higher among children born vaginally than among those delivered by caesarean section, although the difference was not statistically significant (median (25th–75th percentiles): vaginal delivery, *n* = 318: 270 (179–385) ng/mL; caesarean section, *n* = 27: 215 (137–304) ng/mL; *p* = 0.081). Thus, differences in mode of delivery may have contributed to the difference in the proportion of newborns classified below the threshold.

Taken together, our findings indicate that infant iron status is largely shaped early in life, highlighting the importance of initial ferritin concentrations (i.e., neonatal iron stores) for iron status later during childhood. Among the perinatal characteristics, infant sex and cord ferritin levels were most consistently associated with ferritin concentrations at four and twelve months of age. Further, maternal ferritin levels at four months were associated with children’s ferritin levels at both four and twelve months. Cord ferritin concentrations, together with infant sex, may therefore contribute to early identification of infants at risk of low iron stores. However, neither cord ferritin nor maternal ferritin is routinely assessed in Swedish clinical practice. Although cord blood can be collected at birth without invasive procedures, clinical use of ferritin-based risk assessment would require clinically relevant cut-offs and clear follow-up strategies. Whether this approach, or routine postpartum sampling of mothers as a potentially less intrusive alternative, could translate into clinical preventive strategies warrants further investigation.

This study has several strengths, including the prospective design with repeated ferritin measurements across infancy. The repeated sampling during the first year of life—a critical period for brain development with rapid synapse formation and myelination [42]—provides novel longitudinal data from apparently healthy infants. Also, detailed data on potential explanatory factors of ferritin status was collected, including maternal ferritin concentrations four months postpartum. However, some limitations must be considered, including the lack of ferritin and hemoglobin measurements during pregnancy and at delivery. Therefore, maternal iron deficiency and anemia during pregnancy could not be assessed, although these factors may have affected cord ferritin concentrations. Also, C-reactive protein (CRP) could not be analyzed in this study because the external laboratory was unable to analyze it in EDTA plasma; therefore, children were excluded based on recent parent-reported infection, although this approach may have missed less obvious infections or inflammation. However, ferritin concentrations were generally higher among the excluded children and mothers, suggesting that the parent-reported infection, at least to some extent, may have captured infection and/or inflammation at the time of sampling. In addition, hemoglobin concentrations were not available for this study, and clinically defined iron deficiency anemia could therefore not be evaluated. Furthermore, complementary feeding practices, including intake of iron-rich and iron-fortified foods, dietary supplements, and dietary factors that may enhance or inhibit iron absorption, were beyond the scope of the present study. Such factors may contribute to children’s iron status, particularly at one year of age. However, their relevance at birth and four months is likely limited, as families in Sweden are recommended to introduce only small taste samples (approximately 1 mL) from four to six months of age, provided that these do not interfere with breastfeeding [43]. In the NICE cohort, meat, fish, and other iron-rich foods were generally introduced from six months of age onwards. These associations, as well as associations between ferritin concentrations and later outcomes such as cognitive abilities, will be investigated in future publications after detailed descriptive information on diet and the dietary assessment methods has been published (Stråvik et al., *under review*). Information on growth trajectories during infancy was not available for the investigated time points, although this would be a relevant outcome to investigate in future studies. In addition, statistical power and precision may have been affected by variation in the number of available ferritin measurements across time points and by the relatively low number of children with available breastfeeding information during the first month of life. Further, our cohort consisted predominantly of healthy, term-born infants from mothers who were slightly older and more highly educated than other women delivering at the same hospital during the same time period [44], which may limit the generalizability of our results. Although characteristics were generally similar between included and excluded children in this study, excluded children had slightly shorter breastfeeding duration, a higher prevalence of caesarean section deliveries, and lower maternal education. Therefore, selection bias due to missing ferritin data or exclusions related to infection cannot be ruled out.

## 5. Conclusions

In this cohort of healthy Swedish children and their mothers, ferritin concentrations declined successively across the first year of life. Infant sex, cord ferritin concentrations, and maternal postpartum ferritin levels were independently associated with infant ferritin concentrations across the first year of life, whereas neither breastfeeding nor formula feeding was associated with ferritin concentrations in the present analyses. Although cord ferritin concentrations were associated with infant ferritin concentrations later in infancy, cord ferritin is not routinely assessed in Swedish clinical practice. Cord blood can be collected at birth without invasive procedures, but whether cord ferritin can be used to identify infants at risk of low iron stores during infancy requires further investigation. Furthermore, since maternal ferritin at four months postpartum was associated with infant ferritin at both four and twelve months, maternal sampling may offer a less intrusive alternative to repeated infant sampling. However, the clinical relevance and potential use of maternal ferritin as a proxy for infant iron stores remain to be elucidated.

## Figures and Tables

**Table 1:** Background characteristics of all included mother–infant dyads (*n* = 434).

	Available, *n*	Median (25th–75th Percentiles)
Gestational age (days)	433	281 (275–288)
Weight, child		
Birth (g)	431	3565 (3265–3955)
One year (kg)	388	10 (9–11)
Height, child		
Birth (cm)	421	50 (49–52)
One year (cm)	385	76 (74–78)
Breastfeeding duration (months) ^1^	400	8 (6–11)
Formula feeding duration (months) ^1^	400	6.5 (0–10)
Age, mother (years)	434	30 (27–34)
BMI, mother (kg/m^2^) ^2^	424	24.3 (22.0–28.3)
	**Available, *n***	***n* (%)**
Sex (boy)	434	201 (46)
Cesarean section	434	46 (11)
Preterm birth	433	22 (5)
Small-for-gestational-age ^3^	431	
<10th percentile		33 (8)
<2 SD		8 (2)
Large-for-gestational-age ^3^	431	
>90th percentile		60 (14)
>2 SD		16 (4)
Education, mother	431	
Elementary school, 9 years		7 (2)
High school, 12 years		116 (27)
Higher education, > 12 years		308 (71)

Abbreviations: BMI, body mass index; SD, standard deviation. ^1^ Includes zeros for those who responded to the questionnaires but responded that they did not breastfeed/give formula at all. The number displayed as duration (e.g., 8 indicates that the last month of breastfeeding/formula was reported in month eight covering the past month). ^2^ At admission to the maternity ward in early pregnancy. ^3^ Based on ultrasound-derived, sex-specific growth curves [24].

**Table 2:** Ferritin concentrations (ng/mL) during the first year of life.

			Below Thresholds		
	*n*	Median (25th–75th Percentiles)	Cut-Off ^1^	*n*	% (95% CI)
**Child**					
Birth	345	267 (177–382)	<40 ng/mL	12	3.5 (1.9, 5.8)
			<65 ng/mL	24	7.0 (4.6, 10.0)
4 months	133	146 (82–235)	<20 ng/mL	3	2.3 (0.6, 5.9)
12 months	158	30 (19–62)	<10 ng/mL	5	3.2 (1.2, 6.8)
			<12 ng/mL	12	7.6 (4.2, 12.5)
**Mother**					
4 months	133	39 (18–76)	<15 ng/mL	16	12 (7.3, 18.4)

Confidence intervals for proportions were calculated using the binomial method. ^1^ Derived from reference values used clinically and as discussed by clinicians working in a Swedish setting [16,32,33].

**Table 3:** Linear regression models of log_2_-transformed ferritin concentrations and covariates.

	Ferritin Concentration (log_2_-Transformed)								
	*n*	Birth		*n*	4 Months		*n*	12 Months	
		β (95% CI)	*p*		β (95% CI)	*p*		β (95% CI)	*p*
**Infant ferritin concentrations (log_2_-transformed, ng/mL)**									
**Birth**									
Crude	-	-	-	92	0.318 (0.102, 0.533)	0.004	92	0.286 (0.073, 0.500)	0.009
Model 2	-	-	-	92	0.301 (0.089, 0.513)	0.006	92	0.293 (0.081, 0.505)	0.007
Model 3	-	-	-	92	0.292 (0.089, 0.494)	0.005	92	0.306 (0.096, 0.516)	0.005
**4 months**									
Crude	-	-	-	-	-	-	53	0.281 (0.028, 0.535)	0.030
Model 2	-	-	-	-	-	-	53	0.274 (0.021, 0.527)	0.034
Model 3	-	-	-	-	-	-	52	0.198 (−0.077, 0.473)	0.154
**Maternal ferritin at 4 months (log_2_-transformed, ng/mL)**									
Crude	-	-	-	133	0.329 (0.176, 0.482)	<0.001	53	0.259 (0.020, 0.497)	0.034
Model 2	-	-	-	133	0.306 (0.154, 0.457)	<0.001	53	0.239 (−0.004, 0.482)	0.054
Model 3	-	-	-	132	0.333 (0.194, 0.473)	<0.001	52	0.308 (0.078, 0.538)	0.010
**Male sex**									
Crude	345	−0.263 (−0.498, −0.027)	0.029	133	−0.561 (−0.958, −0.163)	0.006	158	−0.567 (−0.902, −0.232)	0.001
Model 2	-	-	-	-	-	-	-	-	-
Model 3	345	−0.274 (−0.510, −0.038)	0.023	132	−0.658 (−1.033, −0.282)	0.001	157	−0.609 (−0.935, −0.282)	< 0.001
**Caesarean section**									
Crude	345	−0.214 (−0.653, 0.225)	0.338	133	−1.186 (−1.780, −0.591)	<0.001	158	−0.149 (−0.649, 0.351)	0.557
Model 2	345	−0.176 (−0.615, 0.262)	0.430	133	−1.064 (−1.660, −0.468)	0.001	158	−0.136 (−0.621, 0.348)	0.579
Model 3	345	−0.166 (−0.605, 0.272)	0.457	132	−1.000 (−1.559, −0.441)	0.001	157	−0.076 (−0.548, 0.397)	0.751
**Preterm birth**									
Crude	345	−0.926 (−1.479, −0.373)	0.001	132	−2.087 (−3.229, −0.946)	<0.001	157	−0.794 (−1.62, 0.033)	0.060
Model 2	345	−0.931 (−1.481, −0.381)	0.001	132	−2.087 (−3.194, −0.980)	<0.001	157	−0.736 (−1.535, 0.063)	0.071
Model 3	345	−1.308 (−2.051, −0.566)	0.001	132	−1.271 (−2.484, −0.057)	0.040	157	−0.029 (−1.14, 1.081)	0.958
**Large-for-gestational-age**									
Crude	343	−0.332 (−0.654, −0.011)	0.043	132	0.478 (−0.183, 1.140)	0.155	157	0.480 (−0.070, 1.030)	0.087
Model 2	343	−0.325 (−0.645, −0.005)	0.047	132	0.577 (−0.067, 1.221)	0.078	157	0.399 (−0.135, 0.933)	0.142
Model 3	343	−0.322 (−0.642, −0.001)	0.049	132	0.496 (−0.111, 1.104)	0.108	157	0.413 (−0.112, 0.937)	0.122
**Gestational age (weeks)**									
Crude	345	0.033 (−0.036, 0.102)	0.350	132	0.253 (0.122, 0.383)	<0.001	157	0.105 (0.018, 0.192)	0.018
Model 2	345	0.040 (−0.029, 0.109)	0.259	132	0.272 (0.146, 0.399)	<0.001	157	0.109 (0.025, 0.193)	0.011
Model 3	-	-	-	-	-	-	-	-	-
**Birth weight (per 100 g)**									
Crude	343	−0.007 (−0.028, 0.015)	0.529	132	0.060 (0.022, 0.097)	0.002	157	0.043 (0.013, 0.074)	0.005
Model 2	343	−0.003 (−0.025, 0.018)	0.762	132	0.069 (0.033, 0.105)	<0.001	157	0.049 (0.020, 0.078)	0.001
Model 3	343	−0.013 (−0.039, 0.014)	0.342	132	0.038 (−0.005, 0.080)	0.081	157	0.041 (0.004, 0.079)	0.030

Exposures were selected based on FDR-adjusted *p* value < 0.05 from Spearman correlation analysis presented in Figure 5. Model 2: Adjusted for sex (binary, 0 = female, 1 = male). Model 3: Adjusted for sex (binary, 0 = female, 1 = male) and gestational age (continuous, weeks).

## Data Availability

Explicit consent to deposit raw data was not obtained from the participants. The R code for the statistical analyses can however be obtained from: https://gitlab.com/miastravik/.

## Supplementary Materials

The following supporting information can be downloaded at: https://www.mdpi.com/article/10.3390/nu18111657/s1. Supplementary Table S1: Characteristics of included mother–infant dyads and those with available ferritin who were excluded due to infection or missing infection information; Supplementary Table S2: Characteristics of included mother–infant dyads and those excluded due to missing ferritin data, reported infection, or missing infection information.; Supplementary Table S3: Ferritin concentrations (ng/mL) during the first year of life among included observations and observations excluded due to reported or unknown infection status at the time of sampling; Supplementary Table S4: Plasma ferritin concentrations by breastfeeding extent and formula feeding extent; Supplementary Figure S1: Longitudinal analysis of plasma ferritin concentrations over the first year of life, stratified by sex.
