# Supplementary material for: Longitudinal Plasma Ferritin in the First Year of Life in Relation to Maternal Status, Birth Characteristics, and Breastfeeding
Source: Nutrients. 2026 May 22;18(11):1657. doi: 10.3390/nu18111657 (PMC13257953; doi:10.3390/nu18111657)
Supplement: Supplementary file 1 [file nutrients-18-01657-s001.zip › nutrients-4307466-supplementary.pdf]

nutrients

MDPI

Supplementary Material

Longitudinal Plasma Ferritin in the First Year of Life in Relation to Maternal Status, Birth Characteristics, and Breastfeeding

Mia Stråvik [1,†], Inger-Cecilia Mayer Labba [1,†,*], Malin Barman [1], Linnéa Svärd [1], Nathalie Scheers [1], Anna Sandin [2], Agnes E. Wold [3] and Ann-Sofie Sandberg [1]

[1] Department of Life Sciences, Food and Nutrition Science, Chalmers University of Technology, 412 96 Gothenburg, Sweden; mia.stravik@chalmers.se (M.S.); malin.barman@chalmers.se (M.B.); lsvard@student.chalmers.se (L.S.); nathalie.scheers@chalmers.se (N.S.); ann-sofie.sandberg@chalmers.se (A-S.S.)

[2] Department of Clinical Science, Pediatrics, Sunderby Research Unit, Umeå University, 901 87 Umeå, Sweden; anna.sandin@umu.se

[3] Department of Infectious Diseases, Institute of Biomedicine, Sahlgrenska Academy, University of Gothenburg, 405 30 Gothenburg, Sweden; agnes.wold@microbio.gu.se

* Correspondence: cecilia.mayer.labba@chalmers.se

† These authors contributed equally to this work.

Content

Supplementary Table S1. Characteristics of included mother–infant dyads and those with available ferritin who were excluded due to infection or missing infection information.

Supplementary Table S2. Characteristics of included mother–infant dyads and those excluded due to missing ferritin data, reported infection, or missing infection information.

Supplementary Table S3. Ferritin concentrations (ng/mL) during the first year of life among included observations and observations excluded due to reported or unknown infection status at the time of sampling.

Supplementary Table S4. Plasma ferritin concentrations by breastfeeding extent and formula feeding extent.

Supplementary Figure S1. Longitudinal analysis of plasma ferritin concentrations over the first year of life, stratified by sex.

Supplementary Table S1. Characteristics of included mother-infant dyads and those with available ferritin who were excluded due to infection or missing infection information.

	Included (*n* = 434)		Excluded (*n* = 56)		
	**Available, *n***	**Median (25th–75th percentiles)**	**Available, *n***	**Median (25th–75th percentiles)**	***p*** [4]
Gestational age (days)	433	281 (275-288)	55	282 (274-287)	0.824
Weight, child					
Birth (g)	431	3565 (3265-3955)	54	3583 (3271-3996)	0.578
One year (kg)	388	10 (9-11)	43	10 (9-11)	0.308
Height, child					
Birth (cm)	421	50 (49-52)	54	51 (49-52)	0.346
One year (cm)	385	76 (74-78)	43	76 (74-79)	0.632
Breastfeeding duration (months) [1]	400	8 (6-11)	39	8 (4-10)	0.154
Formula feeding duration (months) [1]	400	6.5 (0-10)	39	2 (0-8)	0.098
Age, mother (years)	434	30 (27-34)	56	30 (28-35)	0.336
BMI, mother (kg/m^2) [2]	424	24.3 (22.0-28.3)	56	24.0 (21.6-27.9)	0.830
	**Available, *n***	***n* (%)**	**Available, *n***	***n* (%)**	***p*** [5]
Sex (boy)	434	201 (46)	56	26 (46)	1.000
Cesarean section	434	46 (11)	56	12 (21)	0.027
Preterm birth	433	22 (5)	55	0 (0)	0.157
Small-for-gestational-age [3]	431		54		
< 10th percentile		33 (8)		4 (7)	1.000
< 2 SD		8 (2)		0 (0)	0.607
Large-for-gestational-age [3]	431		54		
> 90th percentile		60 (14)		5 (9)	0.404
> 2 SD		16 (4)		2 (4)	1.000

Supplementary Table S1 (continued)

Education, mother	431		56		
Elementary school, 9 years		7 (2)		3 (5)	0.164 (0.205 [6])
High school, 12 years		116 (27)		16 (29)	
Higher education, > 12 years		308 (71)		37 (66)	

Abbreviations: BMI, body mass index; SD, standard deviation.

[1] Includes zeros for those who responded to the questionnaires but responded that they did not breastfeed/give formula at all. The number displayed as duration (e.g., 8 indicates that the last month of breastfeeding/formula was reported in month eight covering the past month). [2] At admission to the maternity ward in early pregnancy. [3] Based on ultrasound-derived, sex-specific growth curves [1]. [4] Differences were tested using Mann-Whitney U test. [5] Differences were tested using Fisher's exact test for binary variables and Pearson's Chi-Square test for multilevel variables. [6] Difference tested using Linear-by-Linear association.

nutrients MDPI

Supplementary Table S2. Characteristics of included mother-infant dyads and those excluded due to missing ferritin data, reported infection, or missing infection information.

	Included (n = 434)		Excluded (n = 195)		
	**Available, n**	**Median (25th–75th percentiles)**	**Available, n**	**Median (25th–75th percentiles)**	**p [4]**
Gestational age (days)	433	281 (275-288)	188	280 (273-286)	0.070
Weight, child					
Birth (g)	431	3565 (3265-3955)	188	3570 (3235-3959)	0.838
One year (kg)	388	10 (9-11)	132	10 (9-11)	0.188
Height, child					
Birth (cm)	421	50 (49-52)	182	50 (49-52)	0.934
One year (cm)	385	76 (74-78)	131	76 (74-79)	0.518
Breastfeeding duration (months) [1]	400	8 (6-11)	123	7 (4-10)	0.027
Formula feeding duration (months) [1]	400	6.5 (0-10)	123	6 (0-9)	0.152
Age, mother (years)	434	30 (27-34)	194	30 (27-34)	0.744
BMI, mother (kg/m^2) [2]	424	24.3 (22.0-28.3)	189	24.4 (22.3-27.1)	0.884
	**Available, n**	**n (%)**	**Available, n**	**n (%)**	**p [5]**
Sex (boy)	434	201 (46)	190	90 (47)	0.862
Cesarean section	434	46 (11)	189	34 (18)	0.013
Preterm birth	433	22 (5)	188	6 (3)	0.400
Small-for-gestational-age [3]	431		187		
< 10th percentile		33 (8)		18 (10)	0.428
< 2 SD		8 (2)		1 (<1)	0.290
Large-for-gestational-age [3]	431		187		
> 90th percentile		60 (14)		27 (14)	0.900
> 2 SD		16 (4)		9 (5)	0.512

Supplementary Table S2 (continued)

Education, mother	431		189		
Elementary school, 9 years		7 (2)		8 (4)	0.036 (0.015 [6])
High school, 12 years		116 (27)		62 (33)	
Higher education, > 12 years		308 (71)		119 (63)	

Abbreviations: BMI, body mass index; SD, standard deviation.

[1] Includes zeros for those who responded to the questionnaires but responded that they did not breastfeed/give formula at all. The number displayed as duration (e.g., 8 indicates that the last month of breastfeeding/formula was reported in month eight covering the past month). [2] At admission to the maternity ward in early pregnancy. [3] Based on ultrasound-derived, sex-specific growth curves [1]. [4] Differences were tested using Mann-Whitney U test. [5] Differences were tested using Fisher's exact test for binary variables and Pearson's Chi-Square test for multilevel variables. [6] Difference tested using Linear-by-Linear association.

Supplementary Table S3. Ferritin concentrations (ng/mL) during the first year of life among included observations and observations excluded due to reported or unknown infection status at the time of sampling.

			Below thresholds		
	n	Median (25th–75th percentiles)	Cut-off [3]	*n*	% (95% CI)
**Child**					
**4 months**					
Without infection [1]	133	146 (82-235)	< 20 ng/mL	3	2.3 (0.6, 5.9)
Infection/unknown [2]	72	172 (104-272)		2	2.8 (0.6, 8.6)
Infection	24	189 (140-256)		0	0.0 (0.0, 9.8)
Unknown	48	152 (97-276)		2	4.2 (0.9, 12.7)
**12 months**					
Without infection	158	30 (19-62)	< 10 ng/mL	5	3.2 (1.2, 6.8)
			< 12 ng/mL	12	7.6 (4.2, 12.5)
Infection/unknown	126	29 (20-51)	< 10 ng/mL	2	1.6 (0.3, 5.0)
			< 12 ng/mL	5	4.0 (1.5, 8.5)
Infection	68	28 (19-48)	< 10 ng/mL	1	1.5 (0.2, 6.7)
			< 12 ng/mL	3	4.4 (1.3, 11.3)
Unknown	58	30 (21-59)	< 10 ng/mL	1	1.7 (0.2, 7.8)
			< 12 ng/mL	2	3.4 (0.7, 10.6)
**Mother**					
**4 months**					
Without infection	133	39 (18-76)	< 15 ng/mL	16	12 (7.3, 18.4)
Infection/unknown	72	44 (23-95)		5	6.9 (2.7, 14.6)
Infection	24	97 (26-129)		0	0 (0.0, 9.8)
Unknown	48	41 (21-68)		5	10.4 (4.1, 21.3)

Confidence intervals for proportions were calculated using the binomial method. [1] Included in the present study. [2] Excluded from the present study. [3] Derived from reference values used clinically and as discussed by clinicians working in a Swedish setting [2–4].

nutrients MDPI

Supplementary Table S4. Plasma ferritin concentrations by breastfeeding extent and formula feeding extent.

Feeding extent	*n*	Median (25th–75th percentiles)	*p* [1]
**Breastfeeding**			
First month			
None	24	220 (117-385)	
Partially	53	258 (178-350)	
Exclusively	181	268 (188-386)	0.587
Fourth month			
None	29	111 (82-235)	
Partially	18	194 (67-259)	
Exclusively	85	146 (83-226)	0.624
Twelfth month			
None	129	31 (19-56)	
Partially	28	26 (18-78)	
Exclusively	0	-	0.647[2]
**Formula feeding**			
First month			
None	169	269 (186-388)	
Partially	71	258 (175-353)	
Exclusively	17	204 (96-362)	0.188
Fourth month			
None	77	142 (83-250)	
Partially	26	165 (88-220)	
Exclusively	28	114 (82-233)	0.770
Twelfth month			
None	115	32 (17-62)	
Partially	36	31 (20-74)	
Exclusively	0	-	0.419

[1] Differences in ferritin concentrations were tested using Kruskal-Wallis test. No differences were observed when comparing exclusively breastfed with no breastfeeding using Mann-Whitney U test (all p values > 0.05).

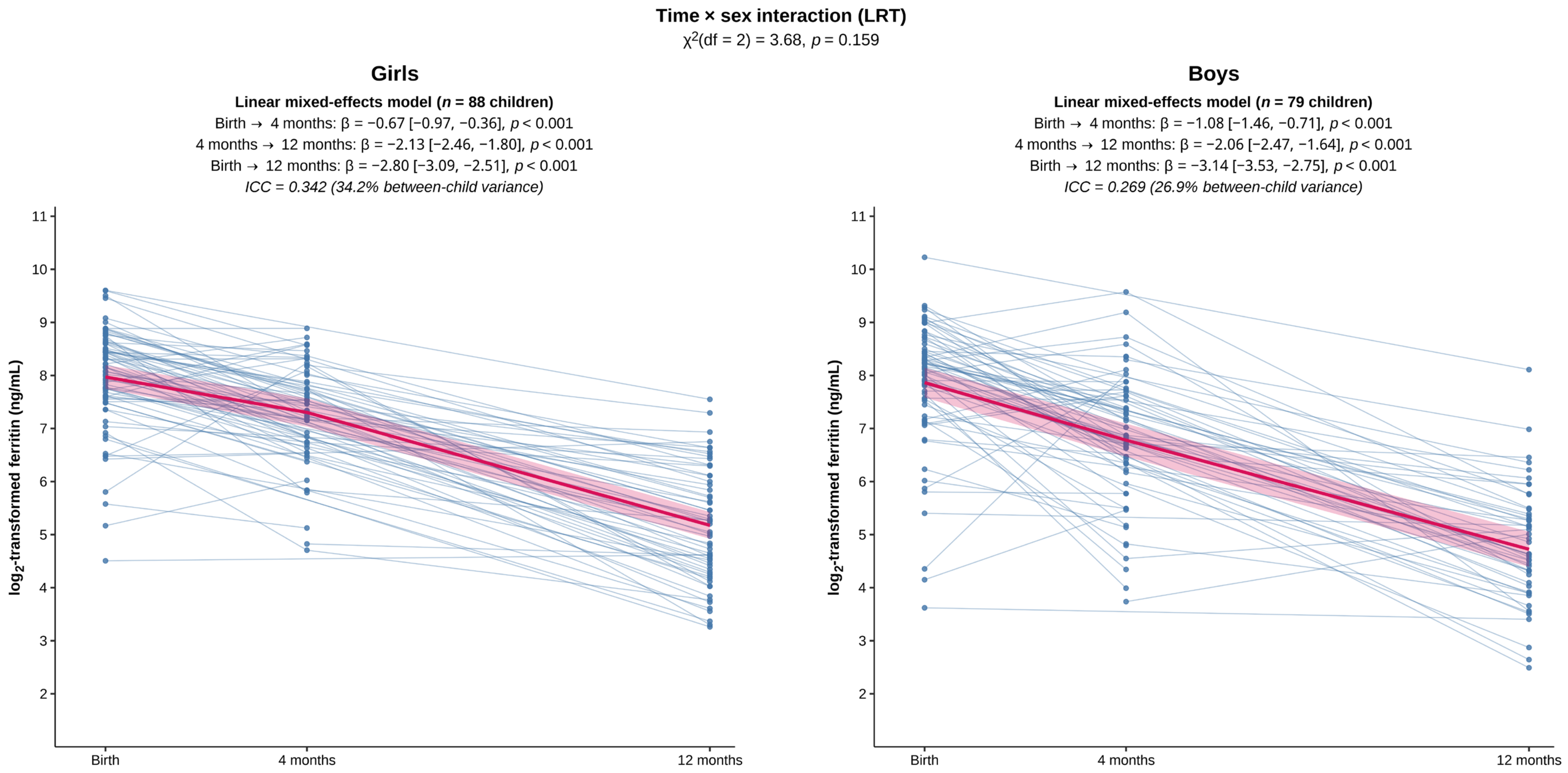

Supplementary Figure S1. Longitudinal analysis of plasma ferritin concentrations over the first year of life, stratified by sex.

Blue lines show individual children's repeated measures; the red line and shaded area indicate the model-based mean ± 95% CI. Regression coefficients for four months versus birth and twelve months versus birth, as well as the model-estimated pairwise difference between twelve and four months, are presented as β. The time × sex interaction was assessed using a likelihood ratio test comparing models with and without the interaction term.

nutrients MDPI

References

1. Maršál, K.; Persson, P.H.; Larsen, T.; Lilja, H.; Selbing, A.; Sultan, B. Intrauterine growth curves based on ultrasonically estimated foetal weights. *Acta Paediatr.* **1996**, *85*, 843–848.
2. Domellöf, M.; Berglund, S.K. *Nutritional anemia in infants and children.* In *Nutritional Anemia*, 2nd ed.; Karakochuk, C.D.; Zimmermann, M.B.; Moretti, D.; Kraemer, K., Eds.; Springer Nature: Cham, Switzerland, **2022**; pp. 77–90.
3. Berglund, S.; Domellöf, M. Barns järnbehov och hur vi bäst kan skydda barnhjärnan. *Läkartidningen* **2014**, *111*, CH9D.
4. Vårdplaneringsgruppen för Pediatrisk Hematologi (VPH). *Järnbristanemi: Vårdriktlinje*, v251111; Barnläkarföreningens delsektion för hematologi och onkologi: Sweden, 2025.
